# A Case Report of Kearns-Sayre Syndrome: Not an Absolute Contraindication for Radiotherapy

**DOI:** 10.7759/cureus.42229

**Published:** 2023-07-21

**Authors:** Shaun Z Yap, Abdul Rahim Mohd Tahir, Thomas P Shakespeare

**Affiliations:** 1 Radiation Oncology, Mid North Coast Cancer Institute Coffs Harbour, New South Wales, AUS

**Keywords:** radiation side effects, radiation response, mitochondrial dna mutation, cm (cutaneous melanoma) bcc (basal cell carcinoma) scc (squamous cell carcinoma), vmat radiotherapy, general radiation oncology

## Abstract

Kearns-Sayre syndrome (KSS) is a rare mitochondrial disorder, and the effects of radiotherapy on such a population group are unknown. A 60-year-old male with a history of KSS was diagnosed with locally advanced basal cell carcinoma along the left inner canthus. He was treated at our institution with curative intent radiotherapy alone and tolerated it well with no major acute or late toxicities. There was a complete clinical and radiological response of the tumor, with no evidence of recurrence 2.5 years after treatment. Further research is needed to explore the effects of ionizing radiation on patients with mitochondrial DNA defects.

## Introduction

Kearns-Sayre syndrome (KSS, OMIM #530000) is a rare multisystem disorder arising from mitochondrial cytopathy [1], usually due to a single large deletion of mitochondrial DNA (mtDNA) [2,3]. The clinical manifestations include chronic progressive external ophthalmoplegia, pigmentary retinopathy, cardiac conduction defects, and cerebellar dysfunction, with the age of onset being <20 years of age [1-3].

Based on early experiments, it has been traditionally thought from a radiobiological perspective that DNA within the nucleus of the cell (nDNA) was the primary target of ionizing radiation (IR) for cell kill [4]. However, there appears to be some emerging evidence that IR can also cause damage to mtDNA, which may have a significant influence on epigenetic control, metabolism, and signaling pathways [5].

The large mtDNA deletions in KSS often include subunits of mtDNA involved in the oxidative phosphorylation pathway [6]. This can be more noticeable in tissues with high energy demand, such as cardiac muscle, kidneys, and brain tissue [6]. It is therefore potentially possible that pre-existing mtDNA deletion from KSS may lead to increased radiation hypersensitivity and increased severity of toxicity due to the potentially increased damaging effects of IR via damage to mtDNA, leading to mitochondrial dysfunction, generation of reactive oxygen species (ROS) and oxidative stress. To our knowledge, there have been no known publications on the biological effects of IR on patients who have KSS, nor on the safety or efficacy of radiotherapy on such a population group.

## Case presentation

Clinical presentation

We report a case of a 60-year-old male who had been clinically diagnosed with KSS at a young age with severe hearing impairment, cardiac conduction defect, as well as ophthalmoplegia and retinopathy, with overall very poor vision in both eyes (legally blind in the left eye). Other current related comorbidities include cardiomyopathy, pacemaker insertion, mitral valve repair, renal impairment, chronic immunosuppression post renal transplant in 1978, multiple excised cutaneous squamous cell carcinomas, subclinical hypothyroidism, and type 2 diabetes (diet-controlled). His regular medications include warfarin, omeprazole, Bactrim, carvedilol, azathioprine, prednisolone, magnesium, rocaltriol, and sertraline.

**Figure 1:**
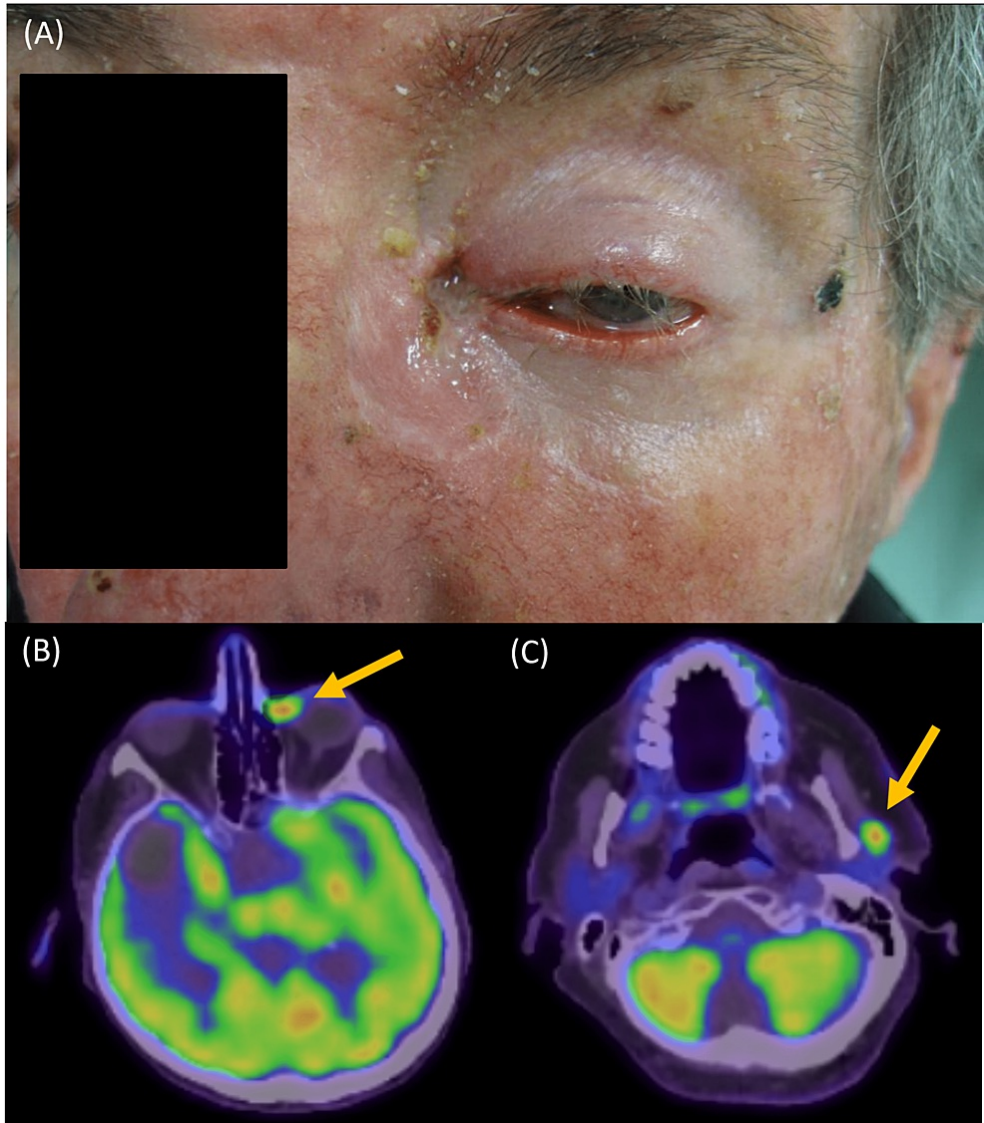
(A) Clinical photograph of the basal cell carcinoma of the skin along the left inner canthus. (B) Composite reconstruction of the FDG-PET-CT scan with axial slices showing intense FDG avidity along the left inner canthus (see arrow), as well as (C) the left parotid gland (see arrow). FDG-PET-CT: Fluorodeoxyglucose-positron emission tomography-computed tomography

He initially presented with a two-year history of a weeping lesion along the left inner canthus. A biopsy confirmed a basal cell carcinoma (BCC). Computed tomography (CT) of the orbit showed the tumor involving the frontal process of the maxilla and the medial aspect of the left globe at the insertion of the medial rectus muscle. Staging CT scan of the head, neck, and chest also incidentally found a right temporal lobe mass, which was subsequently identified on fluorodeoxyglucose (FDG)-positron emission tomography (PET) as a non-tracer avid mass, in keeping with intracranial hemorrhage rather than metastatic neoplasia. The PET scan identified the tracer avid left inner canthus mass, as well as some uptake in the left parotid gland (Figures 1A-1C). MRI of the brain was not performed due to pacemaker incompatibility. Subsequent biopsy of the left parotid lesion was again consistent with infiltrating BCC.

His case was discussed at the Head and Neck Multidisciplinary Team Meeting at a tertiary hospital. Surgery involving an orbital exenteration was considered, but the patient declined due to the morbidity involved. Systemic treatment with vismodegib was also considered, but the patient was unable to obtain private funding for it and there were no available clinical trials or access programs for vismodegib in this particular case. Therefore, the recommended approach was for definitive radiotherapy. This was discussed with the patient, and he provided informed consent to proceed, understanding the potential unknown risks with his history of KSS.

Treatment and outcomes

**Figure 2:**
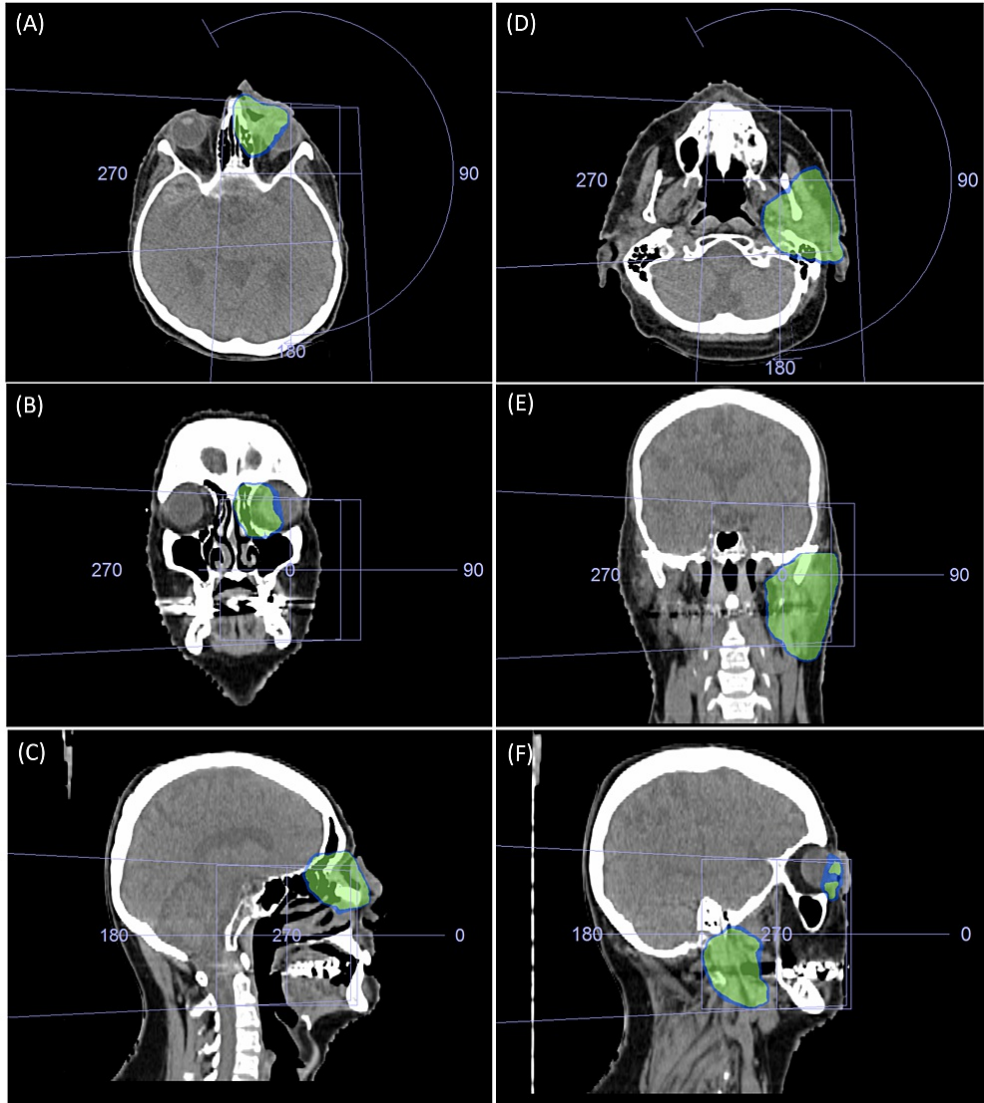
Treatment plan showing 90% (blue) and 95% (green) isodose coverage of the left inner canthus (A: axial view; B: coronal view; C: sagittal view) and left parotid gland (D: axial view; E: coronal view; F: sagittal view) using a Volumetric Arc Radiotherapy (VMAT) technique.

We treated this patient at our institution with curative intent radiotherapy to the left orbit and left the parotid region to a total dose of 60 Gy in 30 fractions given conventionally over six weeks using a Volumetric Arc Radiotherapy (VMAT) technique (Figures 2A-2F). During radiation therapy, he was seen weekly by the treating Radiation Oncologist or Registrar, as well as the dietician, speech pathologist, and nurse in a multidisciplinary Head and Neck Clinic. Cone beam CT (CBCT) was performed daily to ensure adequate soft tissue and bony matching. An ophthalmologist saw him separately on a weekly basis while he was on treatment to assess prospectively if he required a tarsorrhaphy for potential acute dry eye syndrome.

**Figure 3:**
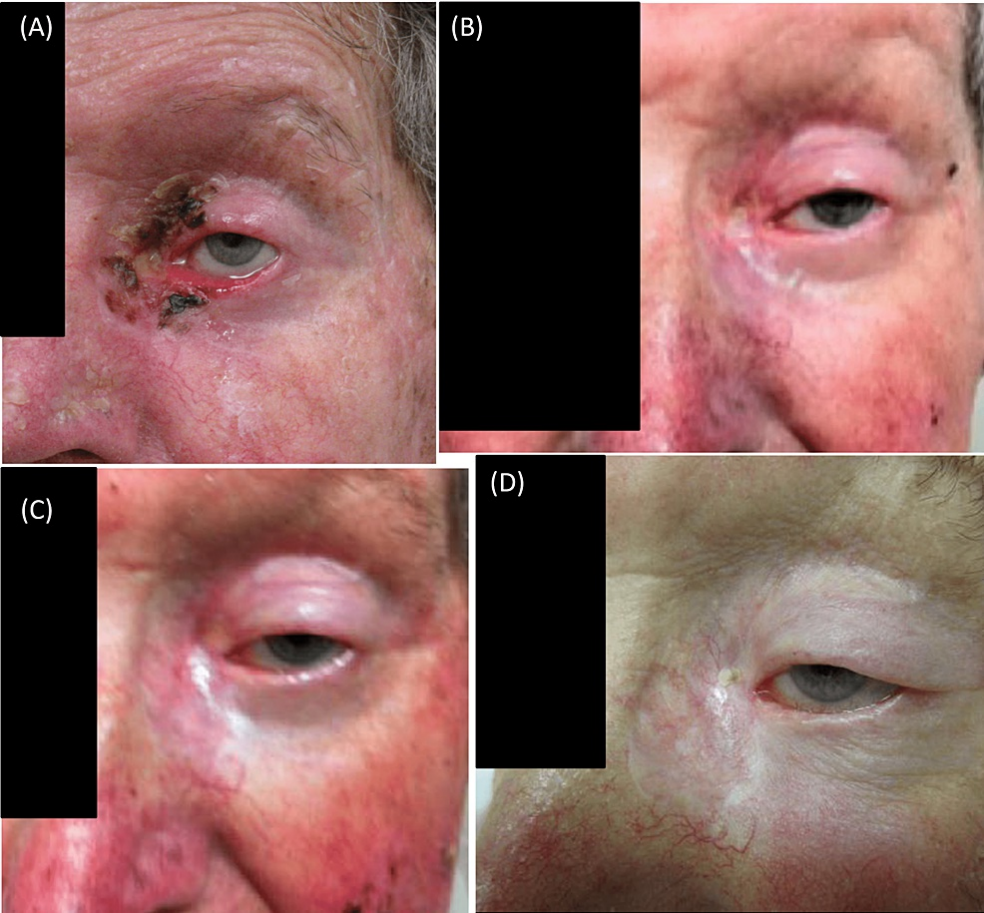
Clinical photographs of the patient at various time intervals post radiation. (A) One week post treatment; (B) three months’ post treatment; (C) six months’ post treatment; and (D) one year post treatment.

He tolerated treatment well, with no requirement for a tarsorrhaphy, and had no weight loss. Toxicities were graded using CTCAE v3.0, and acute toxicities included grade 1 dysgeusia, grade 1 fatigue, and grade 2 radiation dermatitis. Late toxicities included grade 1 epiphora in the left eye and grade 1 epilation of the left eyebrow and eyelashes. There were no acute or long-term complications with corneal ulceration or fibrosis. Post-treatment, he had complete clinical and radiological response at 3 months and there were no signs of local recurrence in the left inner canthus and left parotid. He continued to have regular three- to six-monthly follow-up visits in the outpatient clinic (Figures 3A-3D).

Two and a half years later, he developed a localized cutaneous squamous cell carcinoma (SCC) of the left cheek. In view of his comorbidities and aim for quality of life, the patient opted for palliative radiotherapy and was prescribed 30Gy in six fractions to the left cheek with appositional 9MeV electrons, given twice weekly for three weeks. Toward the end of radiotherapy, he developed a grade 2 radiation dermatitis along his left cheek, corresponding to the treatment field. There were no other significant acute toxicities reported. Unfortunately, a full evaluation of his treatment response to the left cheek was incomplete as he developed a rapid systemic and functional decline around the same time, the cause of which was unclear as he also had possible undiagnosed malignancies with pancreatic and renal masses, which he had opted not to further investigate. Progression of mitochondrial myopathy was thought to be the most likely explanation for his presentation, although this could not be proven. He eventually died in hospital at about 2.5 weeks post-completion of palliative radiotherapy.

## Discussion

KSS is a rare mitochondrial disease, and to our knowledge, there have been no reported cases or series that demonstrate the safety and expected outcomes of delivering radiotherapy to such a population group. We present the first case report of a patient with KSS demonstrating excellent local tumor control with no significant acute or late toxicities from radiotherapy, indicating the possibility that having KSS is not an absolute contraindication for radiotherapy.

Although the pathophysiology of KSS is related to mtDNA mutation, the diagnosis is often made via clinical findings only. The clinical severity of symptoms correlates to the proportion of mutated to normal mtDNA, as KSS is a disease that exhibits mtDNA heteroplasmy [7]. This was the case in our patient, where the diagnosis was made primarily by clinical findings, with significant pre-existing cardiac conduction defects, ophthalmoplegia, and retinopathy. It is potentially possible for the majority of cells within the radiation field to have a higher proportion of normal mtDNA expression, resulting in no significant radiation toxicity beyond expected levels. In addition, we believe that our careful approach to radiotherapy in this situation with good supportive care, conventional fractionation (2Gy per fraction), as well as the use of a highly conformal technique (VMAT), could have contributed to his fairly low toxicity even at curative doses. Table 1 shows a summary of the dose volume statistics for the organs at risk (OARs); these were well within normal dose constraints as according to the Australian EviQ guidelines [8].

**Table 1:** Dose-volume statistics of the patient's treatment plan for the planning target volume (PTV) and organs-at-risk (OARs). PRV: Planning organ-at-risk volume

Structure	Volume (cm^3^)	Min. Dose (Gy)	Max. Dose (Gy)	Mean Dose (Gy)	Ref. Vol. (cm^3^)	Ref. Vol. (%)	Ref. Dose (Gy)
Left Globe	8.318	26.13	60.41	50.18	0.012	0.14	60
					2	24	57.6
					5.1	61	50
					5.9	72	45
					6.7	81	40
					7.6	91	35
					8.2	98	30
					8.318	100	25
Left Lacrimal Gland	0.19	24.23	31.07	27.17	0.007	3.68	30
Left Lens	0.258	55.65	59.61	58.02	-	-	-
Left Occipital Lobe	76.618	6.34	49.29	15.43	0	0	54
Left Optic Nerve	0.644	29.42	52.66	38.78	0	0	55
Left Temporal Lobe	83.79	3.26	44.54	14.58	0	0	54
Brainstem	27.982	56.6	22.77	11.69	-	-	-
Brainstem PRV	52.08	5.37	25.98	12.23	-	-	-
Optic Chiasm	0.998	15.15	21.83	18.22	-	-	-
Spinal Cord	12.894	0.4	20.35	9.42	-	-	-
Spinal Cord PRV	27.364	0.38	21.23	8.8	-	-	-
Mandible	48.366	4.16	62.82	18.41	-	-	-
PTV Left Orbit	31.496	41.35	66.58	60.52	30.281	96	57
PTV Left Parotid	95.444	47.36	65.19	61.3	93.677	98	57
Frontal Lobe	93.858	0.69	58.81	14.84	0.241	0.26	54
Left Inner Ear	0.992	17.73	45.45	28.17	-	-	-
Right Globe	9.138	2.36	6.1	3.7	0	0	35
Right Lens	0.268	2.97	5.08	3.59	0.001	0.37	5
Right Parotid	32.112	3.42	12.03	8.55	-	-	-
Right Optic Nerve	0.776	3.99	20.17	14.99	-	-	-
Right Temporal Lobe	88.618	3.2	16.67	7.84	-	-	-
Oral Cavity	47.356	8.48	29.61	15.9	-	-	-

Another possible reason for low toxicity rates in our patient could be explained by the type of cells affected. We know from DNA mapping that the mtDNA deletions in KSS mainly affect the generation of proteins that form part of the oxidative phosphorylation pathway [2]. Tissues with high energy consumption will have high numbers of mitochondria to carry out oxidative phosphorylation to generate energy, and as a result, they may be more susceptible to IR-induced damage [5]. Such tissues can include muscles (such as cardiac muscle) and brain tissue [5,9], where the high cellular energy demand can easily result in radiation-induced damage and loss of function even with low doses of IR [10]. In our patient, the main areas which received therapeutic doses of IR were the left eye and parotid gland, neither of which were organs of high metabolic demand, hence possibly explaining why the effects seen were not as severe as expected. The radiation field did include a small area of the left temporal lobe of the brain which received a mean dose of 14.6Gy and the brainstem which received a mean dose of 11.7Gy (Table 1). However, these were well within dose constraint limits and did not result in any significant acute or late neurological toxicities in our patient.

The radiobiological effects of IR on mitochondria may perhaps play a role in patients with mitochondrial defects such as KSS, as in this case. Averbeck and Rodriguez-Lafrasse [5] postulate that although mtDNA only comprises 0.25% of total DNA compared to nDNA, the mitochondrial effects from IR can be fairly significant as mtDNA is more vulnerable than nDNA due to its less efficient repair mechanisms and relative lack of protection against oxidative damage. At therapeutic levels of IR exposure, it is thought that the resultant mitochondrial dysfunction and damage can easily induce apoptosis [5,10], which is one of the main mechanisms of tumor cell death. This may be related to the effect of upregulating ROS generation in the tumor cell, which results in growth inhibition and promotion of the apoptotic pathway [5,11]. Current experimental data suggest that targeting mitochondria can result in improved radio-responsiveness in tumor cells [12]. Given that there is already an existing degree of mitochondrial dysfunction in patients with KSS, it could be theorized that radiation damage to mtDNA in these patients can result in an additive effect in inducing apoptosis, thus improving the tumor control probability. Our patient did have an excellent clinical and radiological response, but more research is obviously needed to prove this hypothesis.

## Conclusions

In summary, to our knowledge, this is the first reported case of a patient with KSS who has been successfully treated with radiotherapy, with excellent clinical response and fairly low toxicity. In that regard, careful planning with shared decision-making with the patient and good supportive care is recommended in such a population group. Further research is needed to validate our experience and explore the effects of radiotherapy in patients with KSS who have an oncological diagnosis, with caution and attention given to particular organs at risk including the heart and brain.
